# Ocular Surface Microbiota in Contact Lens Users and Contact-Lens-Associated Bacterial Keratitis

**DOI:** 10.3390/vision5020027

**Published:** 2021-06-03

**Authors:** Jasmine Andersson, Josef K. Vogt, Marlene D. Dalgaard, Oluf Pedersen, Kim Holmgaard, Steffen Heegaard

**Affiliations:** 1 Department of Ophthalmology, Rigshospitalet-Glostrup, University of Copenhagen, 2600 Glostrup, Denmark; kimholmgaard1@gmail.com (K.H.); sthe@sund.ku.dk (S.H.); 2 The Novo Nordisk Foundation Center for Basic Metabolic Research, University of Copenhagen, N 2200 Copenhagen, Denmark; josef.korbinian.vogt@sund.ku.dk (J.K.V.); oluf@sund.ku.dk (O.P.); 3 Department of Health Technology, Technical University of Denmark, 2800 Kongens Lyngby, Denmark; marld@dtu.dk

**Keywords:** ocular surface microbiota, conjunctival microbiota, opportunistic pathogens, contact lenses, bacterial keratitis, contact-lens-associated bacterial keratitis, cultivation, 16S rRNA gene amplicon sequencing

## Abstract

Our objectives were to investigate whether the conjunctival microbiota is altered by contact lens wear and/or bacterial keratitis and to explore the hypothesis that commensals of conjunctival microbiota contribute to bacterial keratitis. Swab samples from both eyes were collected separately from the inferior fornix of the conjunctiva of non-contact-lens users (n_participants_ = 28) and contact lens users (n_participants_ = 26) and from patients with contact-lens-associated bacterial keratitis (n_participants_ = 9). DNA from conjunctival swab samples was analyzed with 16S rRNA gene amplicon sequencing. Pathogens from the corneal infiltrates were identified by cultivation. In total, we identified 19 phyla and 283 genera; the four most abundant genera were *Pseudomonas*, *Enhydrobacter*, *Staphylococcus*, and *Cutibacterium*. Several pathogens related to bacterial keratitis were identified in the conjunctival microbiota of the whole study population, and the same bacteria were identified by both methods in the conjunctiva and cornea for four patients with contact-lens-associated bacterial keratitis. The overall conjunctival microbiota profile was not altered by contact lens wear or bacterial keratitis; thus, it does not appear to contribute to the development of bacterial keratitis in contact lens users. However, in some individuals, conjunctival microbiota may harbor opportunistic pathogens causing contact-lens-associated bacterial keratitis.

## 1. Introduction

Microbial keratitis is caused by microorganisms, such as bacteria and fungi, and is a sight-threatening condition globally affecting up to 2 million people per year [1]. Contact lens wear is the major predisposing risk factor for microbial keratitis [2,3,4]. Ocular surface microbiota have been suggested to play a role in the maintenance of the local ocular homeostasis and protective immunity against infections, such as microbial keratitis [5]. Alterations of ocular surface microbiota have been associated with several ophthalmic diseases such as conjunctivitis, dry eye disease and blepharitis [5]. Thus, it is feasible to assume that the reported alterations to ocular surface microbiota through contact lens wear [6,7] may also contribute to the development of microbial keratitis [8]. Shin et al. [6] demonstrated a more skinlike microbiota in contact lens users with higher relative abundances of *Methylobacterium*, *Lactobacillus*, *Acinetobacter*, and *Pseudomonas* and lower abundances of *Haemophilus*, *Streptococcus*, *Staphylococcus*, and *Corynebacterium*. Zhang et al. [7] observed similar bacterial diversity in contact lens users compared with non-contact-lens users, decreased relative abundances of *Delftia*, *Bacillus*, *Tatum* and *Lactobacillus* and increased levels of *Elizabethkingia*. To our knowledge, no previous studies have investigated the potential alterations of the conjunctival microbiota profile in contact-lens-associated bacterial keratitis (CLABK) using molecular techniques. However, conjunctival microbiota has been described for fungal keratitis by Ge et al. [9], who reported that the conjunctival microbiota profile was altered with lower bacterial diversity and with differences in the relative abundances for some bacteria such as *Corynebacterium*, *Staphylococcus* and *Pseudomonas*.

The bacterial pathogen(s) for CLABK may originate from contact lens biofilms or contact lens cases/solutions [10,11,12,13,14]. Alternatively, CLABK may be the result of an opportunistic infection caused by resident commensals of the normal conjunctiva [5]. *Pseudomonas aeruginosa* is one of the most common causative bacterial pathogens for bacterial keratitis [1,2,15,16,17], and several previous studies have identified *Pseudomonas* in normal conjunctival microbiota [6,18,19,20,21,22,23]. Thus, *Pseudomonas* may act as an opportunistic bacterial pathogen in CLABK. Other pathogens commonly identified in CLABK are *Staphylococcus species* [16,24], *Serratia species* [1,16,24], *Stenotrophomonas maltophilia* [1,16], and *Klebsiella pneumoniae* [1,16,24]. Another frequent pathogen of bacterial keratitis is *Cutibacterium acnes* (formerly known as *Propionibacterium acnes)* [25,26]. The genera *Staphylococcus* [18,20,21,22,27,28], *Stenotrophomonas* [18,20], *Klebsiella* [28], and *Cutibacterium* [18,20,21,22,27,28] have all been identified in normal ocular surface microbiota and may similarly be opportunistic pathogens in patients with bacterial keratitis. 

In the present study, we aim to characterize the conjunctival microbiota in contact lens users with and without bacterial keratitis to explore whether contact lenses and/or bacterial keratitis alter the conjunctival microbial profile. Second, we investigate if the conjunctival microbiota harbors bacteria that are causative to bacterial keratitis in contact lens users.

## 2. Materials and Methods

### 2.1. Study Population

Three study groups were included: non-contact-lens users (NCL), contact lens users without bacterial keratitis (CL), and contact lens users with bacterial keratitis (i.e., CLABK). The CL group was recruited from the Department of Ophthalmology Rigshospitalet-Glostrup, Copenhagen, Denmark. Patients with CLABK and without prior antibiotic treatment that attended the Emergency Department of Ophthalmology at Rigshospitalet-Glostrup, Copenhagen, Denmark were included. The control group’s samples (i.e., NCL) were reanalyzed from our previous study [29]. Exclusion criteria for the NCL and CL group were, use of local or systemic antibiotics within 3 months, use of artificial tears or eye drops in general, known ocular surface disease, and previous ocular surgery. All participants in the NCL and CL groups were evaluated by slit lamp to exclude those with abnormalities in the cornea, conjunctiva and eyelids. 

The study was approved by the Scientific Ethics Committee for the Capital Region of Denmark (H-16017300) and written consent was given to all participants at the time of enrollment. We followed the Tenets of Helsinki declaration. All study participants were >18 years of age.

### 2.2. Sample Collection

The inferior fornix of the conjunctiva of both eyes of each participant was sampled three to four times, without the use of local anesthesia, using a sterile DNA- and RNase-free flocked swab (^®^Puritan); patients with CLABK were sampled from the fellow eye without keratitis (CLABK_fellow) and the eye with keratitis (CLABK), respectively. The conjunctival swab samples were stored with lysis buffer in a −80 °C freezer. Additionally, the patients with CLABK were sampled from the corneal infiltrates with an e-Swab and the samples were cultivated at the Department of Microbiology in Rigshospitalet, Copenhagen, Denmark. 

### 2.3. Conjunctival DNA-Isolation, PCR and 16S rRNA Gene Amplicon Sequencing

We applied the same experimental procedures as we did in a recent study [29]. We followed the manufacturer’s instructions to extract DNA with the NucleoSpin Tissue XS kit (Macherey-Nagle, Düren, Germany). The V3 and V4 regions of the 16S rRNA gene were amplified according to the Illumina manual protocol and sequenced with Illumina MiSeq using paired-end 2 × 300-bp reads. Furthermore, in the sequencing step, lanes were spiked with mock samples from ZymoBIOMICS^®^.

### 2.4. Cultivation of Samples of the Corneal Infiltrate

The cultivation procedure followed the Department of Ophthalmology Rigshospitalet’s standard protocol for analyses of keratitis samples. e-Swabs of the corneal infiltrates were vortexed for 20 s, swabs were removed, and the fluid of the samples was spread onto six growth media; 1× anaerobic agar, 1× chocolate blood agar, 1 × 5% horse blood agar, 1× blue agar, 1× serum bouillon, and 1× thioglycollate broth. Anaerobic agar was incubated at 38 °C for five days and observed for growth after 2 and 5 days of incubation. Chocolate agar and 5% horse blood agar were incubated at 38 °C in a CO_2_ incubator and observed for growth after 2 days of incubation. Blue agar was incubated at 37 °C in atmospheric pressure. The enriched Thioglycollate Broth and Serum Bouillon were incubated at 38 °C in ambient conditions and observed for growth after 2 and 5 days of incubation. Identification of the bacterial species was performed using the MALDI-TOF-MS method [30].

### 2.5. Bioinformatics and Statistical Analysis

We used the DADA2 [31] R package for bioinformatic analysis, including quality filtering and trimming of reads. The Decipher [32] R package and SILVA database were applied for taxonomy assignment. The prevalence method in the decontam [33] R package at the amplicon sequence variant (ASV) level was used for contaminant filtration with the same stepwise procedure as in our previous study [29], using three different negative controls (buffer reagents, unused swabs and PCR-reagents) and a classification threshold of *p* = 0.5. We removed ASVs with <0.00001% of the total relative abundance and rarefied to 2868 sequencing reads per sample. 

The bacterial diversity of the conjunctival microbiota was estimated with Shannon diversity indexes (an ecological measure based on the number of ASVs (species richness) and their proportional abundance in the samples), and with observed richness (the number of ASVs per sample) using the Vegan [34] R package. The Phyloseq [35] R package with the Bray–Curtis dissimilarity index was used to estimate the dissimilarities of the bacterial communities of the samples based on the number of ASVs per sample (i.e., dissimilar vs. similar bacterial communities). Nonmetric multidimensional scaling (NMDS) with the Bray–Curtis dissimilarity index visualized the differences in bacterial composition in two dimensions and PERMANOVA was used as a statistical test to detect potential significant dissimilarities. 

To address the issue with correlated data (i.e., conjunctival samples from both eyes of each participant were included in the data analyses), one randomized eye for each participant of the NCL and CL group was included in the analyses for the comparison of differences between groups (i.e., NCL/CL/CLABK and NCL/CL/CLABK_fellow), with the samples from the patients with CLABK treated as two separate groups in the group comparisons (i.e., CLABK_fellow and CLABK). The Kruskal–Wallis test was used for the comparison of groups NCL/CL/CLABK_fellow and NCL/CL/CLABK, and the Wilcoxon signed-rank test was employed for the analyses of potential differences of eye samples of the same participant/patient. The bacterial taxonomy analysis was reported as the relative abundance of identified bacteria, which refers to the proportion of the bacteria in each sample. We computed the analyses in R (v. 3.6.0) and used the false discovery rate (FDR) to correct multiple testing for detecting differences in relative abundance; *p*-values were considered significant at <0.05. Nonparametric statistical tests were used due to the non-normal distribution of data and the unequal sample sizes of the groups. We reported the median values and interquartile range (IQR) for age, sex, bacterial diversity, and relative abundances considering the non-normal distribution of data used for statistical analyses. 

## 3. Results

In total, 63 participants were included in the study: NCL (*n* = 28), CL (*n* = 26), and patients with CLABK (*n* = 9) (Table 1). Conjunctival swab samples from both eyes from each participant/patient in the NCL (*n* = 56), CL (*n* = 52), and CLABK (*n* = 18) groups yielded a total number of 126 samples for analysis with 16S rRNA gene amplicon sequencing (Table 1). Additionally, cultivation results from the eye with corneal infiltrates (CLABK) were obtained (*n* = 9) (Table 1). The median ages were 32 years (IQR 28–35) in the NCL group, 33 years (IQR 26–38) in the CL group and 44 years (interquartile range (IQR 32–52) for the patients with CLABK. The gender distribution was 50% females and 50% males in the NCL group, 61% females and 39% males in the CL group, and 56% females and 44% males for patients with CLABK (Table 1). The 16S rRNA gene amplicon sequencing analysis of 126 conjunctival samples and 34 negative controls yielded 5,673,653 merged high-quality sequencing reads with an average of 35,023 reads per sample. After removal of 429 ASVs from the contaminant filtration analysis, 40,186 ASVs remained. After rarefaction, 5165 ASVs were left, resulting in the annotation of 19 phyla and 283 genera. For CLABK samples and cultivation of potential pathogens, only five out of nine corneal swab samples (56%) showed growth with more than one bacterial species. 

### 3.1. 16S rRNA Gene Amplicon Sequencing

#### 3.1.1. Bacterial Diversity and Composition of Ocular Surface Microbiota for NCL versus CL/CLABK_fellow

**Figure 1:**
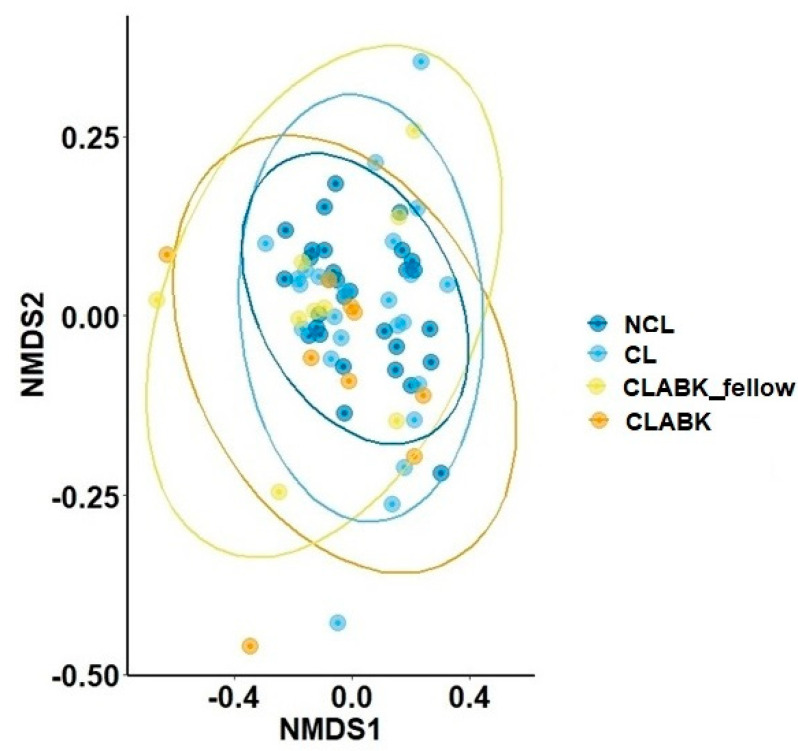
Comparison of the bacterial composition of ocular surface microbiota using the Bray–Curtis dissimilarity index for the study population. The bacterial composition was compared at the amplicon sequence variant (ASV) level and visualized in a two-dimensional plot with nonmetric multidimensional scaling (NMDS). Conjunctival samples from both eyes of the patients with contact-lens-associated bacterial keratitis (eye with bacterial keratitis (CLABK) and the eye without bacterial keratitis (CLABK_fellow)) were compared with one randomized eye for each participant in the following groups: contact lens users without bacterial keratitis (CL) and non-contact-lens users (NCL). The bacterial composition was not significantly different between groups (pairwise comparison with PERMANOVA); NCL vs. CL *p* = 1.0; NCL vs. CLABK_fellow *p* = 0.49; NCL vs. CLABK *p* = 0.35; CL vs. CLABK_fellow *p* = 1.0; CL vs. CLABK *p* = 0.82. Stress ratio = 0.14.

The median Shannon diversity indexes were similar for the NCL group compared with CL/CLABK_fellow: NCL (4.6, IQR 4.3–5.0), CL (4.5, IQR 4.1–5.0), and CLABK_fellow (3.9, IQR 3.8–4.5; *p* = 0.48; Table 1). The highest median of observed richness at the ASV level was observed for NCL (297, IQR 247–335) and lower numbers were observed for CL/CLABK_fellow (281, IQR 220–337/251, IQR 174–281, respectively); however, no statistically significant difference was demonstrated (*p* = 0.56, Table 1). The median number of genera was similar for all groups (NCL *n* = 30 (IQR 24–33), CL *n* = 25 (IQR 23–30) and CLABK_fellow *n* = 27 (IQR 19–29); *p* = 0.52; Table 1). The analysis of the bacterial composition showed an overall clustering pattern in all groups. Hence, the bacterial communities resembled each other, and this observation was statistically confirmed (pairwise PERMANOVA, *p* > 0.05; Figure 1). 

#### 3.1.2. Bacterial Diversity and Composition of Ocular Surface Microbiota for NCL/CL versus CLABK

Shannon diversity index (median 4.8, IQR 3.6–5.2) and the observed richness (ASV and genus level) of the conjunctival CLABK samples (median 314, IQR 206–359 and median 28, IQR 19–34, respectively) were similarly compared with NCL and CL (*p* = 0.48 and *p* = 0.40, respectively; Table 1). A clustering pattern of the samples’ bacterial composition was observed for CLABK, NCL, and CL groups, which indicated that the bacterial communities of these groups were similar (PERMANOVA, *p* > 0.05; Figure 1).

#### 3.1.3. Bacterial Taxonomy of Ocular Surface Microbiota from the Whole Study Population

**Figure 2:**
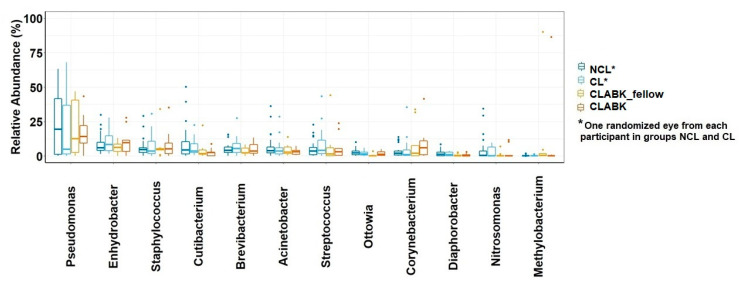
Relative abundance (%) of the 12 most common genera of ocular surface microbiota in the whole study population. Boxplots for relative abundance with medians and interquartile range (IQR) are shown for non-contact-lens users (NCL), contact lens users without bacterial keratitis (CL), fellow eye of patients with contact-lens-associated bacterial keratitis (CLABK_fellow) and for eyes with bacterial keratitis (CLABK). Data from one randomized eye of each participant in the NCL and CL groups were compared with data from CLABK_fellow and CLABK. No significant difference was demonstrated with the Kruskal–Wallis test (p_adj_ > 0.05) for NCL vs. CL/CLABK_fellow or for NCL vs. CL/CLABK.

The median relative abundance of the 12 most common genera for the whole study (respectively, NCL, CL, CLABK_fellow, and CLABK) were *Pseudomonas* (19.5%; 4.9%; 12.6%; 14.1%), *Enhydrobacter* (6.0%; 8.2%; 6.3%; 9.6%), *Staphylococcus* (4.6%; 3.4%; 4.5%; 5.0%), *Cutibacterium* (4.3%; 3.4%; 1.8%; 2.4%), *Brevibacterium* (4.0%; 5.4%; 2.3%; 3.6%), *Acinetobacter* (3.8%; 3.6%; 2.7%; 3.2%), *Streptococcus* (3.5%; 4.1%; 1.4%; 3.0%), *Ottowia* (2.2%; 1.5%; 0.1%; 1.1%), *Corynebacterium* (1.9%; 1.0%; 2.0%; 5.9%), *Diaphorobacter* (1.1%; 0.9%; 0.2%; 0.04%), *Nitrosomonas* (0.4%; 0%; 0%; 0%), and *Methylobacterium* (0%; 0%; 0.3%; 0%) (Figure 2). For NCL compared with CL/CLABK_fellow, no significant difference was demonstrated for these 12 genera (p_adj_ > 0.05), nor were CLABK samples significantly different in comparison with NCL and CL (p_adj_ > 0.05). We further explored the potential difference of the relative abundances of these 12 genera at an individual level, i.e., the conjunctival samples of the same participant/patient were compared within the groups (NCL, CL and patients with CLABK (i.e., CLABK_fellow versus CLABK)). No significant difference was demonstrated for the 12 genera (Wilcoxan signed-rank test, p_adj_ > 0.05; data not shown). 

#### 3.1.4. Several Genera Were Present in the Ocular Surface Microbiota from CLABK_fellow and CLABK of the Same Patient 

**Figure 3:**
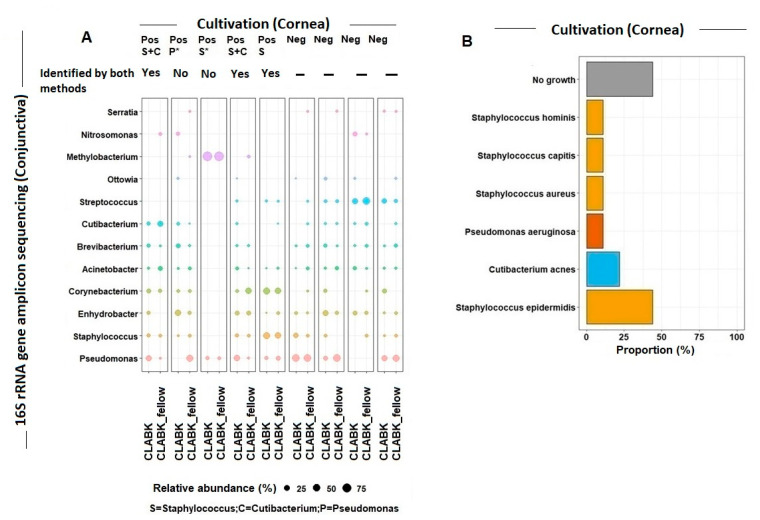
(**A**) 16S rRNA gene amplicon sequencing versus cultivation of the ocular surface microbiota. For contact lens users with bacterial keratitis (i.e., CLABK = eye with bacterial keratitis; CLABK_fellow = eye without bacterial keratitis), the relative abundance for the most common genera identified in the conjunctiva using 16S rRNA gene amplicon sequencing was compared with the cultivation results from the sampling of the corneal infiltrates. Five cultivations were positive for growth (Pos), defined as ≥one bacterial species, and four cultivations were without growth (Neg). The potential bacterial pathogens of the cornea were identified at the genus level as *Staphylococcus* (S), *Cutibacterium* (C), and *Pseudomonas* (P). For four out of the five positive cultures, the same bacteria at the genus level were detected in the CLABK samples with 16S rRNA gene amplicon sequencing and by cultivation. (The relative abundance for *Staphylococcus* was ≤1% in one CLABK sample and therefore not shown in the figure (marked with *)). *Pseudomonas* was identified by cultivation in one patient; however, the relative abundance was 0% in the CLABK sample using 16S rRNA gene amplicon sequencing. Minimum relative abundance for the shown genera was 1%. (**B**) Cultivation results of the corneal infiltrates in the CLABK samples. Six bacterial species were identified, and *Staphylococcus epidermidis* was the most frequently cultivated potential pathogen with growth in four out of five patients (80%). One to three bacterial species were cultivated per patient, while cultivations for four out of nine patients (44%) showed no growth.

Two to six genera per patient (genera with a relative abundance ≥ 1%) were identified in both CLABK_fellow and CLABK of the same patient (Figure 3A). The most frequently observed genera represented in CLABK_fellow and CLABK of the same patient were *Acinetobacter* (*n* = 8), *Pseudomonas* (*n* = 7), *Enhydrobacter* (*n* = 7), *Brevibacterium* (*n* = 7), and *Staphylococcus* (*n* = 6) (Figure 3A).

### 3.2. Cultivation versus 16S rRNA Gene Amplicon Sequencing of Ocular Surface Microbiota 

#### Bacteria Identified in the Cornea and Conjunctiva of Patients with CLABK

Cultivation of the swabs of the corneal infiltrates for five out of nine patients showed bacterial growth (56%) for at least one bacterium (Figure 3B). The most frequently identified bacteria by cultivation were of the *Staphylococcus species* (four out of the five positive cultivations (80%)) and observed in samples of corneal infiltrates and conjunctiva (CLABK_fellow and CLABK) (Figure 3A,B). The *Staphylococcus species* identified by cultivation were *Staphylococcus epidermidis*, *Staphylococcus aureus*, *Staphylococcus capitis*, and *Staphylococcus hominis* (Figure 3B). *Staphylococcus epidermidis* was the most frequently identified cultivated pathogen (44%) (Figure 3B). In four out of five positive cultivations, the same corneal microbes (*Staphylococcus* and *Cutibacterium*) were identified at their corresponding genus level in the conjunctival samples (CLABK_fellow and CLABK), except for the fifth patient with confirmed *Pseudomonas aeruginosa* keratitis, for whom *Pseudomonas* was only identified in the conjunctiva of the eye without bacterial keratitis (CLABK_fellow) (Figure 3A). 

### 3.3. Opportunistic Pathogens in Conjunctival Microbiota of the Whole Study Population

**Figure 4:**
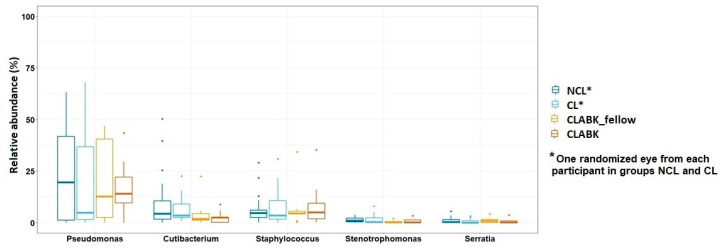
The relative abundance of five known bacterial pathogens to bacterial keratitis. No significant difference of the relative abundance of these five genera was demonstrated for the comparison between non-contact-lens users (NCL) vs. contact lens users (CL)/fellow eye of patient with contact-lens-associated bacterial keratitis (CLABK_fellow) or for the comparison between NCL/CL vs. eyes with bacterial keratitis (CLABK) (Kruskal–Wallis test, *p* > 0.05).

*Pseudomonas*, *Cutibacterium*, *Staphylococcus*, *Stenotrophomonas*, and *Serratia* were all identified at their corresponding genus level in the conjunctival samples of patients with CLABK (CLABK_fellow and CLABK) and in the CL and NCL groups (Figure 4). The relative abundances of these five known pathogens to bacterial keratitis were not significantly different in the comparison of NCL/CL/CLABK_fellow or NCL/CL/CLABK, nor when compared at an individual level in each group (Wilcoxan signed-rank test, *p* > 0.05, data not shown). The conjunctival microbiota was also explored for other common bacterial pathogens such as *Escherichia coli, Klebsiella pneumonia* and *Enterobacter cloacae*; however, these bacterial species were not identified in our study.

## 4. Discussion

The role of ocular surface microbiota in the pathogenesis of ocular infections is sparsely described in the literature [9]. To our knowledge, this is the first study characterizing conjunctival microbiota in CLABK based on 16S rRNA gene amplicon sequencing. Our study investigated whether the conjunctival microbiota was altered by contact lens wear and/or CLABK. No significant difference in the bacterial diversity or composition was demonstrated for the CL group or for patients with CLABK compared with the NCL group. Thus, conjunctival microbiota with contact lens wear and CLABK does not appear to be a risk factor for the development of bacterial keratitis in contact lens users. However, the causative bacterial pathogens in CLABK may originate from harboring commensals in the conjunctival microbiota, rather than from the external environment, as several potential bacterial pathogens (at genus level) were identified in the conjunctival microbiota of the whole study population and in the conjunctiva of both eyes in patients with CLABK. These observations indicate that commensals of the conjunctival microbiota are pathobionts and may act as opportunistic pathogens. 

In agreement with other studies [6,7,23] about the ocular surface microbiota in contact lens users, we identified several of the same most common genera; however, the relative abundances of the most common genera in our study were not significantly different compared to the non-contact-lens users. In contrast to our results, other studies [6,7] have reported differences of the relative abundances of a few genera for contact lens users compared to the non-contact-lens users. Shin et al. [6] reported more skinlike ocular surface microbiota in contact lens users with higher abundances of *Methylobacterium*, *Lactobacillus*, *Acinetobacter* and *Pseudomonas* and lower abundances of *Haemophilus*, *Streptococcus*, *Staphylococcus* and *Corynebacterium* compared with controls. The discrepancy of our results compared with the study by Shin et al. [6] may be explained by differences in sampling timepoints and when the contact lenses were last used. In our study, all contact lens users without bacterial keratitis had interrupted their use of contact lenses for >2 days. Thus, the observed changes by Shin et al. [6] may be a temporary alteration of the conjunctival microbiota in contact lens users, and our results may reflect the normalization of the conjunctival microbiota when contact lenses are not worn for days. 

No previous studies about the conjunctival microbiota in CLABK using 16S rRNA gene amplicon sequencing have been reported. Although, a small recent study of eight patients by Ge et al. [9] reported that the skin-associated microbe *Corynebacterium* was less abundant in patients with fungal keratitis. This is noteworthy, as *Corynebacterium mastitidis* has been shown to be protective against keratitis in mice, and the authors suggested that this bacterium contributes to the pathogenesis of keratitis [36]. *Corynebacterium* was identified in our study as one of the most common genera; however, the relative abundance was similar in all study groups. Ge et al. [9] also observed lower relative abundance of *Staphylococcus* and higher relative abundance for *Pseudomonas* in fungal keratitis compared with controls. We could not confirm this finding in our study. The conflicting results regarding the relative abundance of these three genera reported by Ge et al. [9] and our study may be partly explained by differences in the study design and sample collection. The differences in methodology include types of keratitis investigated (fungal keratitis vs. bacterial keratitis) and severeness of the keratitis (size of infiltrate). Furthermore, we sampled a different type of swab to Ge et al.’s [9] study, with local anesthesia, and only sampled the inferior fornix of the conjunctiva. Overall, we did identify several of the most common genera reported in the study by Ge et al. [9]. 

Our findings indicate that in some cases, bacterial pathogens originating from conjunctival microbiota may be responsible for the infection in patients with CLABK rather than external pathogens, as we identified the same bacterial species of the corneal infiltrate using cultivation and in the conjunctiva at the corresponding genus level using 16S rRNA gene amplicon sequencing. In addition, known pathogens to bacterial keratitis such as *Pseudomonas aeruginosa* [1,15,16,17], *Stenotrophomonas species* [1,16], *Staphylococcus species* [16,24], *Serratia species* [1,16,24], and *Cutibacterium acnes* [25,26] were identified at the genus level in the whole study population, which further confirmed our hypothesis that commensal bacteria in the conjunctiva could be opportunistic pathogens. Ge et al. [9] reported similar results for patients with fungal keratitis and controls [9] regarding the identification of *Pseudomonas*, *Serratia*, and *Staphylococcus* in conjunctival microbiota.

From a clinical perspective, the findings of opportunistic pathogens in normal conjunctival microbiota is notable and one relevant question is as follows: why do some contact lens wearers develop bacterial keratitis whereas others do not? Biofilm formation on contact lenses is presumed to contribute to the increased risk of bacterial keratitis [37]. A higher relative abundance of *Pseudomonas* [9], a known bacterial pathogen to bacterial keratitis [1,2,15,16,17], has been reported for patients with fungal keratitis [9]; however, this finding was not confirmed in our study, nor for other pathogens, e.g., *Staphylococcus* [16,24], *Serratia* [1,16,24], *Stenotrophomonas* [1,16], or *Cutibacterium* [25,26]. Contact lenses disrupt protective mechanisms, such as the intact corneal epithelium, by mechanically causing punctate erosions, thus leaving the cornea more susceptible to infection [38]. The breach of the mechanical barrier of the cornea combined with the presence of opportunistic pathogens in the conjunctival microbiota may contribute to the increased risk of bacterial keratitis in contact lens users.

Limitations to this study include a small overall sample size, unequal sample size between groups, and lack of positive cultivations (56%) from corneal swabs. However, a reasonable high positive cultivation rate was obtained, considering the small-sized infiltrates (thus, limited material sampled) in our study. The reported range of the positive cultivation rate for three previous studies varied between 52.5 and 65% [2,16,39]; Karaca et al. [16] only included corneal infiltrates of ≥3 mm, which yielded 65% positive cultivations. Bacterial keratitis in contact lens users is frequently caused by *Pseudomonas aeruginosa* [17], presumably due to favorable conditions for these microbes, such as the survival niche created through contact lens wear and biofilm formation [1]. We only identified one patient with *Pseudomonas aeruginosa* keratitis, possibly because of the small corneal infiltrates and the small study population of patients with CLABK. Another limitation is the resolution level of taxonomy, where 16S rRNA gene amplicon sequencing in general is not sufficient for the classification of bacteria at the species level; instead, the shotgun sequencing approach could be the preferable choice in future studies because it can classify bacteria at the species or strain level. The two different methods used for the investigation of the conjunctiva and cornea limit the interpretation and comparability of the results; nonetheless, comparison at the genus level is possible using the two methods.

## 5. Conclusions

The conjunctival microbiota profile was similar regarding the bacterial diversity, composition, and relative abundance of the most common genera for contact lens users, patients with contact-lens-associated bacterial keratitis and non-contact lens users. Hence, alterations of the conjunctival microbiota by contact lens wear and in bacterial keratitis in contact lens users do not appear to be a risk factor for the development of bacterial keratitis. However, the conjunctival microbiota may harbor commensals that are pathobionts and may act as opportunistic pathogens causing bacterial keratitis.

## Figures and Tables

**Table 1:** Overview of the study population and alpha diversity analysis results with Shannon diversity index and observed richness. NCL—non-contact-lens users. CL—contact lens users. CLABK_fellow = eye without contact-lens-associated bacterial keratitis; CLABK = eye with contact-lens-associated bacterial keratitis: IQR—Interquartile range; ASV—amplicon sequence variant.

	NCL	CL	CLABK_fellow and CLABK	*p*-Values
**Number of participants**	28	26	9	-
**Age (years) median (IQR)**	32 (28–35)	33 (26–38)	44 (32–52)	2.2 × 10^−16 1^
**Sex (%) (F/M)**	50%/50%	61%/39%	56%/44%	-
**Number of samples**	56 (left and right eye)	52 (left and right eye)	18 (*n* = 9 eye CLABK_fellow and *n* = 9 eye CLABK)	-
**Number of positive cultures from corneal swabs**	NA	NA	5 (56%)	-
**Shannon diversity index** Median (IQR) *	4.6 (4.3–5.0)	4.5 (4.1–5.0)	CLABK_fellow 3.9 (3.8–4.5) CLABK 4.8 (3.6–5.2)	0.48 ^2^ 0.48 ^3^
**Observed richness for ASV level** Median (IQR) *	297 (247–335)	281 (220–337)	CLABK_fellow 251 (174–281) CLABK 314 (206–359)	0.56 ^2^ 0.40 ^3^
**Observed richness for genus level** Median (IQR) *	30 (24–33)	25 (23–30)	CLABK_fellow 27 (19–29) CLABK 28 (19–34)	0.52 ^2^ 0.45 ^3^

* For the NCL and CL groups, one randomized eye was included to calculate the Shannon diversity index and observed richness. ^1^ Kruskal–Wallis test. ^2^ Comparison of NCL vs. CL vs. CLABK_fellow with the Kruskal–Wallis test. ^3^ Comparison of NCL vs. CL vs. CLABK with the Kruskal–Wallis test.

## Data Availability

The data that support the findings of this study can be available from the corresponding author upon reasonable request.
